# CIDEB promotes lipid deposition in goat intramuscular adipocytes

**DOI:** 10.5713/ab.24.0584

**Published:** 2025-02-27

**Authors:** Zhuohan Huang, Qi Li, Changheng Yang, Changhui Zhang, Lian Huang, Yaqiu Lin, Yong Wang, Hua Xiang, Jiangjiang Zhu

**Affiliations:** 1 Key Laboratory of Qinghai-Tibetan Plateau Animal Genetic Resource Reservation and Utilization, Southwest Minzu University, Ministry of Education, Chengdu, China; 2 Qinghai-Tibetan Plateau Animal Genetic Resource Reservation and Utilization Key Laboratory of Sichuan Province, Southwest Minzu University, Chengdu, China; 3 Institute of Qinghai-Tibetan Plateau, Southwest Minzu University, Chengdu, China

**Keywords:** *CIDEB*, Goat, Intramuscular Preadipocytes, Lipid, Lipid Deposition

## Abstract

**Objective:**

Cell death-inducing DNA fragmentation factor alpha-like effector B (*CIDEB*), a family member of Cell death-inducing DFF45-like effectors (CIDEs), is well known as a crucial regulator for lipid metabolic signaling pathways in various metabolic tissues and secretory glands. However, its role in regulating intramuscular fat (IMF) deposition in goat remains unclear.

**Methods:**

The expression vector pcDNA3.1-*CIDEB* was constructed and transfected into goat intramuscular preadipocytes; the overexpression and interference efficiency and expression of genes related to lipid metabolism were measured by Real-time polymerase chain reaction; the effect of overexpression of *CIDEB* and interfering with *CIDEB* on lipid droplet formation was observed by Oil Red O staining and glycerol phosphate oxidase-Trinder enzymatic reaction. Then RNA-Seq was used to investigate the metabolic pathway of *CIDEB* affecting adipocyte deposition in goat intramuscular preadipocytes.

**Results:**

Overexpression of *CIDEB* significantly promoted the lipid droplets accumulation and the triglyceride deposition, and significantly upregulated the expression of genes related to lipid metabolism. After overexpression of *CIDEB* in goat intramuscular preadipocytes, 171 differentially expressed genes (DEGs) were found, including 122 up-regulated and 49 down-regulated DEGs, and the top three significantly changed pathways filtered by Kyoto Encyclopedia of Genes and Genomes (KEGG) analysis were Cocaine addiction, Amphetamine addiction and Malaria pathways. Conversely, the silencing of *CIDEB* significantly reduced lipid accumulation in goat intramuscular preadipocytes, meanwhile changing the expression of lipid metabolism genes. For *CIDEB* silencing, a total of 2140 DEGs were found, including 1252 up-regulated and 888 down-regulated DEGs, and the top three significantly changed pathways filtered by KEGG analysis were Ribosome, Thyroid hormone signaling pathway and Alzheimer disease.

**Conclusion:**

The expression of *CIDEB* can significantly promote lipid deposition of intramuscular adipocytes in goats, and these results provide important data to support further clarifying the mechanism of *CIDEB* gene on the regulation of intramuscular adipogenesis, and the IMF formation in goats.

## INTRODUCTION

Adipose tissue, composed mainly of adipocytes, is a metabolically heterogeneous endocrine organ [1], mainly related to lipid metabolism, energy homeostasis, support structure and meat quality [2]. Anatomically, adipose tissue could be classified as subcutaneous adipose tissue (SAT), visceral adipose tissue (VAT), abdominal adipose tissue (AAT), intramuscular adipose tissue, intermuscular adipose tissue, heart adipose tissue, and kidney adipose tissue [1]. Of these, intramuscular fat is essential for improving the flavor and palatability of meat. Fat deposition traits are genetically and environmentally influenced and can affect meat quality, growth rate and energy metabolism in livestock. However, the molecular mechanism underlying fat deposition in goats is not fully understood [1].

The family of Cell death-inducing DFF45-like effectors (CIDEs) includes CIDEA, *CIDEB*, and CIDEC/Fsp27, named for their homology to the N-terminal domain of DNA fragmentation factor 45 (*DFF45*) [3,4]. CIDEs were firstly found to be associated with apoptosis [3,5,6], and were proved to induces caspase-independent cell death in human [7,8]. Besides apoptosis, CIDEs were also found to involve in cellular lipid metabolism regulation [9], including lipid storage, secretion and synthesis [10,11] in metabolic tissues and secretory glands [12–14], and also plays an important role in the development of obesity, insulin resistance, and fatty liver [15–17]. Several CIDE proteins have also been shown to affect intracellular lipid metabolism, such as lipid metabolism in adipocytes, hepatocytes, and macrophages [18]. Compared with other family members, Cell death-inducing DNA fragmentation factor alpha-like effector B (*CIDEB*) is relatively stable and unaffected by nutritional conditions [19,20], mainly expressed in liver [14] and lower in intestine [21].

The role of *CIDEB* in promoting very low-density lipoprotein (VLDL) lipidation, maturation and secretion has been well studied in mice [22,23]. The absence of *CIDEB* resulted in reduced VLDL lipidation and maturity and altered hepatic cholesterol homeostasis [22,24,25]. The cholesterol and low-density lipoprotein in the blood of *CIDEB*^−/−^ mice were significantly decreased, and the synthesis pathway of cholesterol in the liver was inhibited by *CIDEB* deficiency [25]. What’s more, *CIDEB* also promotes hepatic lipid storage through fusion and growth of hepatic lipid droplets [26]. In mouse liver, CIDEB was found to anchor SREBP/SCAP complex to Coatomer Complex II to regulate the activity of the sterol regulatory element binding protein 1 (SREBP1). CIDEB is also involved in maintaining lipid homeostasis by promoting the loading of the SREBP/SCAP protein complex to the endoplasmic reticulum (ER) exit site and helping its export from the ER [23]. The deficiency of *CIDEB* significantly reduced the activity of *SREBP1*, decreased the expression of downstream target genes of SREBP pathway, and alleviated diet-induced hepatic steatosis. Taken together, these studies performed on CIDEB^−/−^ mice suggest that CIDEB plays a multifunctional role in controlling hepatic lipid secretion, lipid storage, and lipid synthesis. In the early stages of fat formation, the expression of *CIDEB* gene is significantly upregulated, which may contribute to the initiation of fat formation [27].

However, little data was available for the direct association of *CIDEB* gene with adipocyte deposition in domestic animals. In the present study, CIDEB was found to significantly promote cellular lipid deposition by overexpression of *CIDEB* in goat intramuscular preadipocytes. And then transcriptional profiles accompanying the alteration of *CIDEB* was performed to screen the signal pathways induced by *CIDEB* by RNA-Seq in affecting adipocyte deposition. These data may enhance our understanding about the regulation of formation of intramuscular fat in goat.

## MATERIALS AND METHODS

### Cell isolation, culture and treatment

All animal procedures used in this study were approved by the Institutional Animal Care and Use Committee, Southwest Minzu University (permit number: S2020-013, revised on 1 June 2004). Goat primary intramuscular preadipocytes were isolated using the previously described method with minor modification [28]. Briefly, the longissimus dorsi muscle tissues were collected from two-day-old Jianzhou goats from Jianyang County (Sichuan, China) after slaughter. The tissues were then washed 3 times with phosphate-buffered saline (PBS), separated and trimmed on a super clean bench, following which 2 volumes of type II Collagenase (Sigma, St. Louis, MO, USA) was supplemented for digestion at 37°C for 90 min (shaking every 5 min). The digestion was stopped by adding an equal volume of complete culture medium containing 10% fetal bovine serum (DMEM/F12 containing 10% FBS, 1% antibiotic, and 50 μmol/L oleic acids [Sigma, Tokyo, Japan]). The samples were then filtered using sterile gauze and a 75-μm cell strainer, and the cells were then centrifuged at 2,000 r/min for 5 min. After discarding the supernatant, the red blood cell lysate was used for resuspended the cells, followed by standing for 5 min and centrifuging at 2,000 r/min for 5 min. After resuspending in clear complete culture medium, the cells were seeded into 25 cm^2^ culture flasks for the following cultivation under 5% CO_2_ at 37°C with new complete medium after 2 h. The cell culture medium was changed every two days until the cell density reached 80%. For cell treatment, the cells were passed to the third generation at a ratio of 1 to 3 and inoculated into 6-well plate (10-cm^2^). After treatment was completed, the cell culture medium was replaced with an adipocyte-inducing medium.

### Construction of goat *CIDEB* gene overexpression vector and siRNA synthesis

The plasmid of pMD19-T-*CIDEB* was stored in our laboratory. A subcloning system was preformed to amplify the target sequences after added the protective base, Hind III and myc-tagged and BamHI to the primers. The primers as follows: S: CCCAAGCTTATGGAGCAGAAACTCATCTCTGAAGAGGATCTGGAGTACCTCTCTAACCTGGAC, A: CGCGGATCCTCAGTAGGGTTTAAGGCGACCCTG.

Subcloning conditions as follows: 98°C for 3 min; 98°C for 15 s, 57°C for 30 s, 72°C for 2 min, 30 cycles; 72°C for 10 min. The polymerase chain reaction (PCR) product was then recycled and digested and linked to digested pcDNA3.1 to build pcDNA3.1-*CIDEB* recombination plasmid. After being transformed into competent cells DH5α (Tsingke, Beijing, China) and identified by double digestion, the plasmid was validated by DNA sequencing with the help of Chengdu Tsingke biotechnology company.

Negative control (NC) siRNA and two siRNAs of goat *CIDEB* gene were synthesized by Shanghai GenePharma company. siRNA-NC: S: UUCUCCGAACGUGUCACGUTT, A: ACGUGACACGUUCGGAGAATT. siRNA-*CIDEB*165: S: GUCAGGAACUGCUAGACAATT, A: UUGUCUAGCAGUUCCUGACTT. siRNA-*CIDEB*396: S: GCAUCACCUUCGACGUAUATT, A: UAUACGUCGAAGGUGAUGCTT.

### Cell transfection

Cell transfection was performed as previously described [29]. After cell passage, transfection was performed with Lipofectamine 3000 (Thermo Fisher Scientific, Shanghai, China) when the cells filled 70% to 80% of the culture vial. They were transfected with pcDNA3.1-*CIDEB*, pcDNA3.1, siRNA-*CIDEB*165 and NC siRNA. In this experiment, pcDNA3.1-*CIDEB* plasmid group and siRNA-*CIDEB* (si-CIDEB) group were the experimental group, and pcDNA3.1 plasmid (NC) group and negative control siRNA (si-NC) group were the control group. First, the cells are starved; After the medium was discarded and washed three times with PBS, 900 μL Opti medium was added to each well of the six-well plate and placed in a constant temperature incubator at 37°C for 4 hours. After starvation treatment, the transfection premix was prepared: In the overexpression experiment, the premix was 50 μL Opti medium and 3 μL lip3000. Premixed solution B consists of 50 μL Opti medium mixed with 2.5 μL P3000 and 1μg plasmid DNA (pcDNA3.1-*CIDEB* or pcDNA3.1). Mix the premix well and let it sit at room temperature for 20 minutes before using. Interference assay: 100 μL Opti medium, 6 μL lip3000, and 4 μL siRNA were mixed, and the premix was also placed at room temperature for 10 minutes, then gently suspended and instillated to complete cell transfection for overexpression and interference studies. Six hours after transfection, 50 μmol/L Sigma oleic acid induction solution was added, and cells were collected after 48 hours of culture.

### Oil red O staining

Oil red O staining was performed as the previously reported methods [30]. Briefly, the cells were slowly washed 3 times with PBS and fixed with 10% formaldehyde for 30 min. After discarding the formaldehyde and washing the cells 3 times with PBS, Oil Red O working solution (the mixture of 3 mL Oil Red 5 g/L dissolved in isopropanol and 2 mL of ddH_2_O) was supplemented for 20 min incubation at room temperature. Finally, the cells were cleaned with PBS and photographed with inverted fluorescence microscope (Zeiss, Tokyo, Japan). For the quantification of Oil Red O, 1 mL of isopropanol was added to each well of the 6-well plate and the absorbance value at 510 nm was measured using a spectrophotometer (Thermo Fisher Scientific, Shanghai, China).

### Triglyceride determination

Cellular triglyceride content in goat intramuscular adipocytes were used to determine Tissue Triglyceride Content Assay Kit (Applygen, Beijing, China). Briefly, the cells were washed three times with PBS, and treated by 200 μL of triglyceride lysate on ice for 10 min. Then the lysate was divided into two portions for optical density value assay, one for triglyceride detection at 562 nm while the other one for cellular total protein detection by bicinchoninic acid assay at 550 nm according to the manual of the kit (Vazyme, Nanjing, China), which was then used for triglyceride content correction.

### Transcriptome analysis of CIDEB goat intramuscular adipocytes

After the treatment of overexpression and siRNA silencing of *CIDEB* for 48h, the cells were harvested and treated by 1 mL Trizol (Takara, Dalian, China) for total RNA extraction. The RNA transcriptome sequencing was performed by Shanghai OE Biotechnology Co, Ltd. Gene Ontology (GO) and Kyoto encyclopedia of genes and genomes (KEGG) analysis were analyzed using oebiotech (https://www.oebiotech.com/index.php?c=show&id=414). All the experiments were replicated three times.

### Quantitative real-time polymerase chain reaction

To further explore the mechanism of *CIDEB* in lipid metabolism, its overexpression and interference were used to detect the effect on the expression of genes related to lipid metabolism. The primers of quantitative real-time PCR (RT-qPCR) are shown in Table 1. Real-time quantitative PCR was performed using three biological replicates and technical triplicates of each cDNA sample, according to the manufacturer’s protocol. Relative expression levels of target genes for gene correction using ubiquitously expressed transcript (*UXT*) as an internal reference. PCR was performed using the same volume of cDNA sample for each gene to record the cycle threshold (Ct) value. PCR experiments were performed with a Bio-Rad CFX96 PCR System using Taq Pro Universal SYBR qPCR Master Mix (Vazyme, Q712-02). PCR reaction system 10 μL, including 5 μL SYBR Green Real-time PCR Master Mix (Vazyme), 0.2 L (10 μM) primers, 1 μL sample and 3.6 μL ddH_2_O. The following cycling conditions were used: 95°C for 3 min to activate the polymerase followed by 40 cycles of denaturation at 95°C for 10 s, annealing at 60°C for 30 s and extension at 72°C for 30 s. Fluorescence signal was obtained at a temperature of 72°C extension.

### Data analysis

RT-qPCR results were statistically analyzed by 2^−ΔΔCt^ method. All test data were set with at least 3 biological replicates. GraphPad Prism 9.0 was used for statistical analysis and plotting. Two-tailed t-test was used for significance identification.

## RESULTS

### Overexpression of *CIDEB* promotes lipid deposition in goat intramuscular preadipocytes

**Figure 1:**
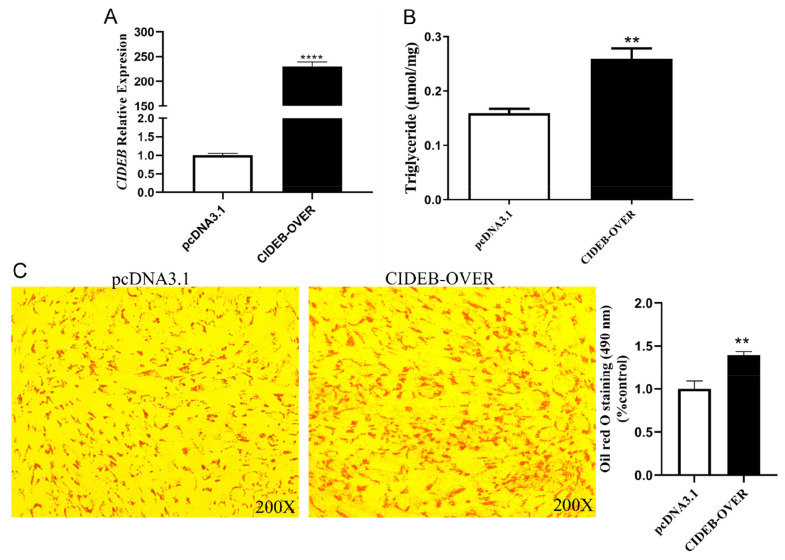
Detection of *CIDEB* overexpression efficiency. (A) Detection of *CIDEB* overexpression efficiency, *CIDEB*-OVER is pcDNA3.1-*CIDEB* treatment group, pcDNA3.1 is control group. (B) Determination of cellular TAG content of *CIDEB* overexpression group and control group. (C) Oil red O staining of intramuscular preadipocytes in control group (pcDNA3.1) and *CIDEB* overexpression group (*CIDEB*-OVER) and Oil red O staining OD value detection (490 nm). ** p<0.01, **** p<0.0001. TAG, triglyceride; OD, optical density.

**Figure 2:**
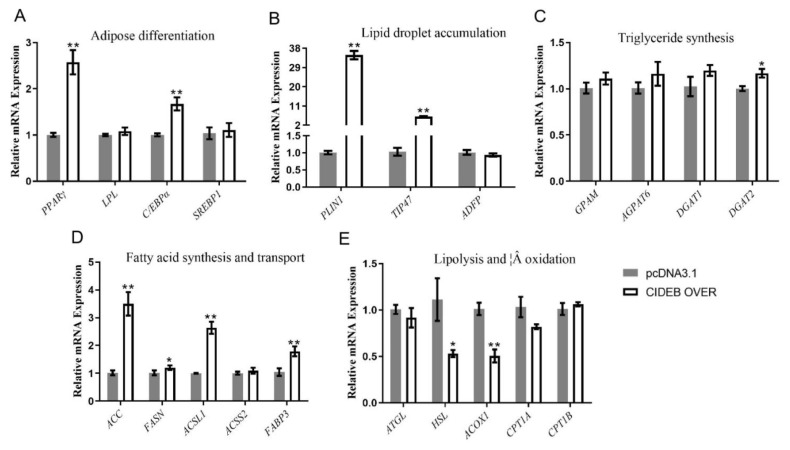
Overexpression of *CIDEB* altered the expression level of related genes. (A) Adipose differentiation related genes (B) Lipid droplet accumulation related genes. (C) Triglycerides (TAG) synthesis related genes. (D) Fatty acid synthesis and transport related genes. (E) Lipolysis and β oxidation related genes. Data are shown as mean±SEM, * p<0.05, ** p<0.01. SEM, standard error of the mean.

To further elucidate the role of *CIDEB* in fat deposition, we ligated the CDS region of the *CIDEB* gene into vector, obtained 5428 bp (pcDNA3.1 vector) and 708 bp (*CIDEB* gene) by Hind III and Bam HI restriction enzymes digestion and transfected them into goat intramuscular precursor adipocytes. The expression level of *CIDEB* was increased by about 230-fold compared with the control after overexpression of *CIDEB* (Figure 1A). The cellular triglyceride was increased significantly by 1.62-fold after overexpression of *CIDEB* compared with control (Figure 1B). Consistent with the results of triglyceride formation, the accumulation of lipid droplets in cells also increased by 1.39-fold compared with control (Figure 1C). Meanwhile, the overexpression of *CIDEB* also significantly increased the expression of adipose differentiation marker genes peroxisome proliferator activated receptor gamma (*PPARγ*) by 1.57-fold and enhancer binding protein alpha (*C/EBPα*) by 1.67-fold (Figure 2A), lipid droplet accumulation-related genes perilipin 1 (*PLIN1*) by 33.82-fold and tail-interacting protein 47 (*TIP47*) by 5.07-fold (Figure 2B), fatty acid synthesis and transport genes acetyl-CoA carboxylase (*ACC*) by 2.48-fold, acyl-CoA synthetase long-chain family member 1 (*ACSL1*) by 1.64-fold, fatty acid binding protein 3 (*FABP3*) by 1.74-fold and fatty acid synthase (*FASN*) by 1.18-fold (Figure 2D), triglyceride synthesis-related gene diacylglycerol O-acyltransferase 2 (*DGAT2*) by 1.16-fold (Figure 2C). Correspondingly, the overexpression of *CIDEB* significantly decreased the expression of lipolysis and fatty acid β oxidation-related genes acyl-CoA oxidase 1 (*ACOX1*) by 50% and hormone-sensitive lipase (*HSL*) by 58% (Figure 2E). In conclusion, it can be considered that overexpression of *CIDEB* promotes adipogenesis in goat intramuscular preadipocytes.

### Effect of *CIDEB*-overexpressing on transcriptome profile in goat intramuscular preadipocytes

**Figure 3:**
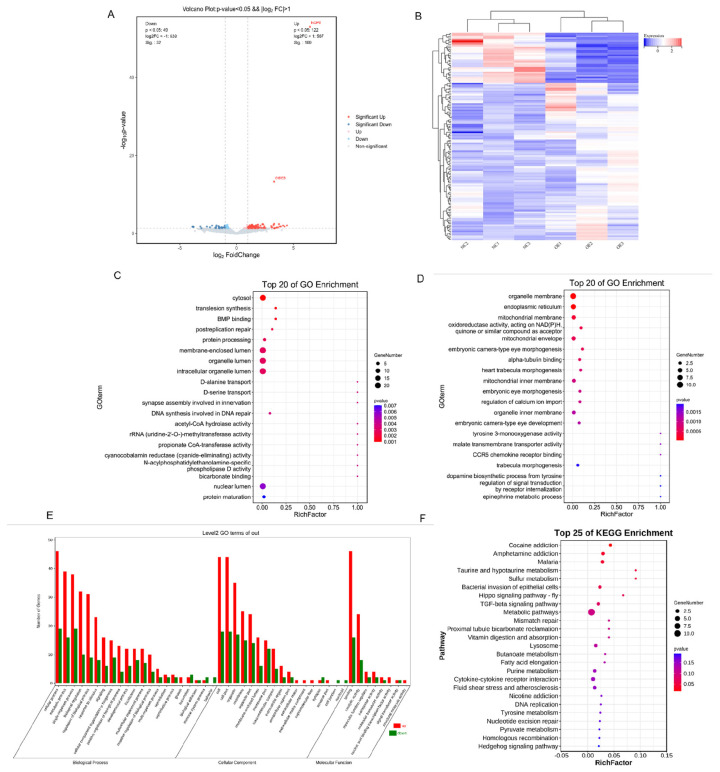
Overexpression of *CIDEB* affected the mRNA transcript profiles. (A) RNA-seq volcano plot of significantly differential expression genes (DEGs) in pcDNA3.1-*CIDEB* (n = 3) vs NC (n = 3) goat intramuscular adipocytes, red and green dots denote upregulated and downregulated genes, respectively. (B) The heat map shows the relative levels of DEGs. (C) GO enrichment analysis of upregulated differential genes. (D) GO enrichment analysis of downregulated differential genes. (E) The GO pathway analysis of related DEGs, up represents up-regulated DEGs, and down represents down-regulated DEGs (F) The KEGG pathway analysis of related DEGs. NC, negative control; GO, Gene Ontology; KEGG, Kyoto Encyclopedia of Genes and Genomes.

**Figure 4:**
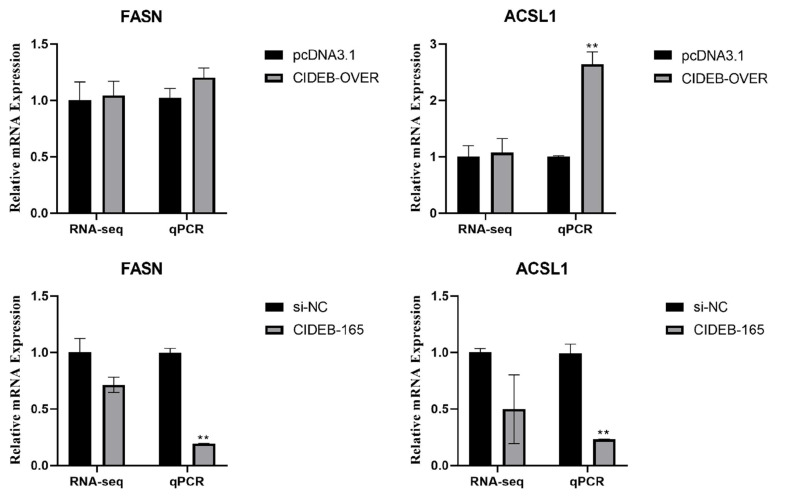
Verification of representative DEGs by qPCR (n = 3), including fatty acid synthase (*FASN*), acyl-CoA synthetase long-chain family member 1 (*ACSL1*). ** p<0.01. DEGs, differential expression genes; NC, negative control; qPCR, quantitative polymerase chain reaction.

To determine the possible mechanism underlying the activated lipid deposition, RNA-Seq analysis was performed after *CIDEB* overexpression in goat intramuscular preadipocytes. In results, a total of 171 differentially expressed genes (DEGs) were screened (p<0.05) by the overexpression of *CIDEB*, including 122 up-regulated genes and 49 down-regulated genes (Figures 3A, 3B). GO enrichment analysis showed that the upregulated DEGs were related to cytosol, membrane-enclosed lumen, organelle lumen, intracellular organelle lumen, nuclear lumen and protein maturation (Figure 3C); the downregulated DEGs were related to organelle membrane, ER, mitochondrial membrane, quinone or similar compound as acceptor, mitochondrial inner membrane and organelle inner membrane (Figure 3D). By GO analysis, the DEGs were enriched in 21 terms of biological processes (mainly containing cellular processes, metabolic processes, biological regulation, biological process regulation and stimulus response), 8 terms of molecular functions (mainly containing binding and catalytic activities) and 17 terms of cellular components (mainly containing cells, cellular structural material, organelles, membrane and organellar structural material) (Figure 3E). Three lipid metabolism-related pathways were screened from the functional annotation results of GO, respectively aminophospholipid transport, lipid metabolic process, and glycerolipid metabolic process (p<0.05) (Table 2). To probe the potential signal pathways by *CIDEB* overexpression, the up- and down-regulated DEGs were analyzed by KEGG analysis respectively. For the up-regulated DEGs, malaria, taurine and hypotaurine metabolism, amphetamine addiction and cocaine addiction were enhanced (Figure 3F). In addition, we verified their expression levels through RT-qPCR analysis. Consistently, overexpression of *CIDEB* downregulated the expression of *FASN* and *ACSL1* (Figure 4).

### Interfering of *CIDEB* inhibited lipid deposition in goat intramuscular preadipocytes

**Figure 5:**
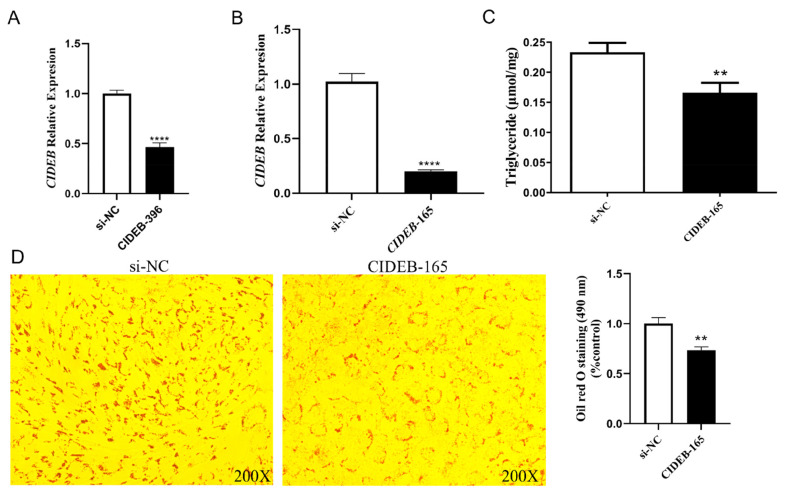
Detection of *CIDEB* interference efficiency. (A) Detection of interference efficiency of *CIDEB*-396. (B) Detection of interference efficiency of *CIDEB*-165. (C)Determination of cellular TAG content of *CIDEB* interference group and control group. (D) Oil red O staining of cells in control group (si-NC) and interference group (*CIDEB*-165) and Oil red O staining OD value detection (490 nm), ** p<0.01, **** p<0.0001. TAG, triglyceride; OD, optical density.

**Figure 6:**
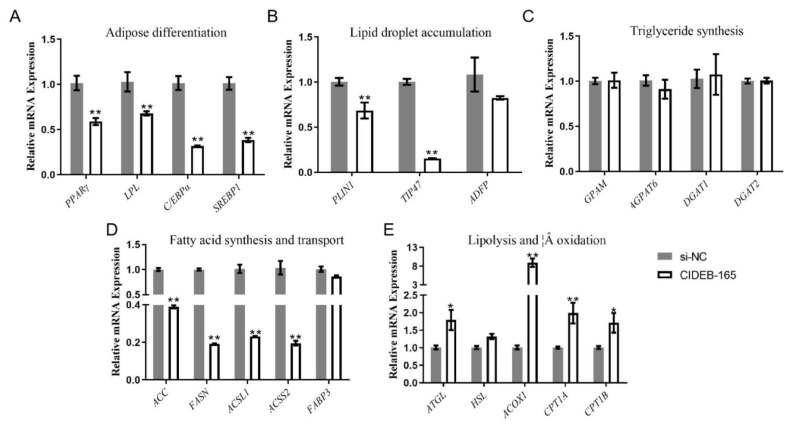
Interference with *CIEDB* altered the expression level of related genes. (A) Adipose differentiation related genes. (B) Lipid droplet accumulation related genes. (C) Triglycerides (TAG) synthesis related genes. (D) Fatty acid synthesis and transport related genes. (E) Lipolysis and β oxidation related genes. Data are shown as mean±SEM, * p<0.05, ** p<0.01. SEM, standard error of the mean.

To elucidate the role of *CIDEB* in regulating intramuscular lipid accumulation in goats, we silenced the expression of *CIDEB* by two pairs of siRNAs in goat intramuscular preadipocytes. The expression level of *CIDEB* was reduced by 82% with treatment of *CIDEB*-165 and 75% with the treatment of *CIDEB*-396 (Figure 5A), so *CIDEB*-165 was used in the subsequent experiment. Contrary to overexpression, the abundance of TAG decreased by 28% after *CIDEB* knockdown (Figure 5B), and the accumulation of lipid droplets decreased significantly by 27% (Figure 5C). The knockdown of *CIDEB* significantly decreased the expression of *PPARγ* by 42%, *LPL* by 35%, *C/EBPα* by 69%, *SREBP1* by 62% (Figure 6A), *PLIN1* by 31%, *TIP47* by 84% (Figure 6B), *ACC* by 62%, *FASN* by 80%, *ACSL1* by 78% and *FABP3* by 14% (Figure 6D), and meanwhile up-regulated the expression of *ATGL* (1.29-fold), *CPT1B* (1.28-fold), *ACOX1* (7.92-fold) and *CPT1A* (1.29-fold) (Figure 6E) by *CIDEB* interference. In conclusion, it can be launched to conclude that interfering of *CIDEB* inhibited lipid deposition in goat intramuscular preadipocytes.

### Effect of *CIDEB*-interference on transcriptome profile in goat intramuscular preadipocytes

**Figure 7:**
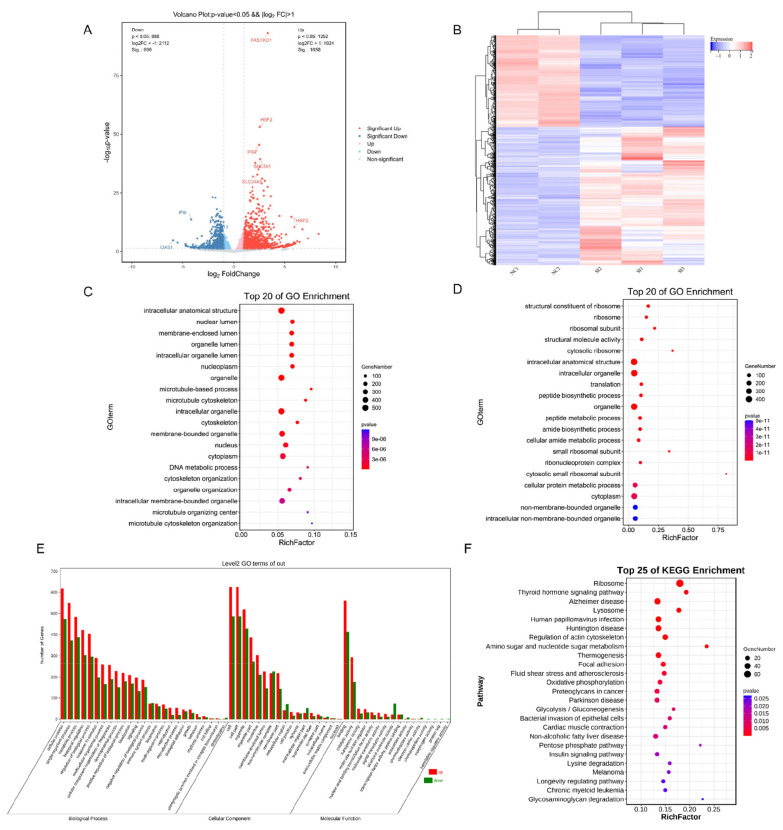
Interference with *CIDEB* affected the mRNA transcript profiles. (A) RNA-seq volcano plot of significantly differential expression genes (DEGs) in SI-*CIDEB* (n = 3) vs NC (n = 3) goat intramuscular adipocytes, red and green dots denote upregulated and downregulated genes, respectively. (B) The heat map shows the relative levels of DEGs. (C) GO enrichment analysis of upregulated differential genes. (D) GO enrichment analysis of downregulated differential genes. (E) The GO pathway analysis of related DEG, up represents up-regulated DEGs, and down represents down-regulated DEGs (F) The KEGG pathway analysis of related DEGs. GO, Gene Ontology; KEGG, Kyoto Encyclopedia of Genes and Genomes; DEGs, differential expression genes; NC, negative control.

To determine the possible mechanism underlying the inactivated lipid deposition, RNA-Seq analysis was performed after *CIDEB* silencing in goat muscle preadipocytes. A total of 2140 DEGs were screened (p<0.05), including 1252 up-regulated genes and 888 down-regulated genes (Figures 7A, 7B). GO enrichment analysis showed that the upregulated DEGs were related to intracellular anatomical structure, organelle, intracellular organelle, membrane-bounded organelle, cytoplasm and intracellular membrane-bounded organelle (Figure 5C); the downregulated DEGs were related to intracellular anatomical structure, intracellular organelle, organelle, cellular protein metabolic process, cytoplasm, non-membrane-bounded organelle, intracellular non-membrane-bounded organelle (Figure 5D). By GO analysis, the DEGs were enriched in 25 terms of biological processes (mainly containing metabolic processes, developmental process, rhythmic process, reproductive process, multi-organism processes, locomotion and growth response), 8 terms of molecular functions (mainly containing binding and catalytic activities) and 17 terms of cellular components (mainly containing organelle part, membrane-enclosed lumen, nucleoid, extracellular region part and extracellular matrix component) (Figure 7E). Four lipid metabolism-related pathways were screened from the functional annotation results of GO, respectively lipid transport involved in lipid storage, lipid storage, aminophospholipid transport and cellular response to lipid (p<0.05) (Table 3). To probe the potential signal pathways by *CIDEB* interference, the up- and down-regulated DEGs were analyzed by KEGG analysis respectively. For the up-regulated DEGs, apelin signaling pathway, insulin signaling pathway, glycolysis/gluconeogenesis and focal adhesion were enhanced (Figure 7F). In addition, we verified their expression levels through RT-qPCR analysis. Consistently, interference with *CIDEB* downregulated expression of *FASN* and *ACSL1* (Figure 4).

## DISCUSSION

We understand that CIDEB as a lipid droplet fusion-associated protein has been extensively studied, especially in mouse and human diabetes, cancer, etc. However, there is still relatively little research on CIDEB in the field of animal husbandry, especially in goats. Although it has an important role in lipid droplet fusion, it does not follow from this that it influences lipid metabolic networks and lipid deposition in goat intramuscular preadipocytes. The innovation of this paper lies in the fact that we systematically verified the regulatory effects of *CIDEB* on intramuscular fat deposition and lipid metabolism networks at both overexpression and interference levels, using a domesticated animal (goat) as the study subject. It is of positive significance for our in-depth understanding of the potential function of CIDEB in goat intramuscular fat formation and its application prospects in animal husbandry from the perspective of molecular breeding.

The crucial role of *PPARγ and SREBP1* in controlling cellular lipid accumulation and the expression of adipogenesis genes have been well studied. In mice, the up-regulated expression of *SREBP1* promoted the lipid fat metabolism and the secretion of VLDL, while the *CIDEB* deficiency cancelled the diet-induced obesity via the significantly decreased of *SREBP1* [14,24]. Even in goats, both of *PPARγ* and *SREBP1* were positively regulated lipogenesis in mammary gland. Recently, the central role of *PPARγ* and its target genes was confirmed in the control of milk fat synthesis in goat mammary epithelial cells [31]. In the present study, the expression of *PPARγ* and *SREBP1* were upregulated by the overexpression of *CIDEB*, and then validated by the decreased expression by *CIDEB* silencing, proposed the hypothesis that CIDEB, as an original function of lipid droplets confusion, may regulate lipid metabolism via the control of PPARγ and SREBP1. Supporting the hypothesis, their downstream genes, including *ACC*, *FASN* and *SCD* et al, were upregulated along with the increased *PPARγ* and *SREBP1.*

Differentiation into adipocytes requires the sequential expression of the transcription factors, C/EBPβ, C/EBPδ, PPARγ, and C/EBPα [32]. C/EBPα plays an important role in cell proliferation, differentiation, metabolism, inflammation, and other responses. Numerous studies have shown that many adipose-specific gene promoters have C/EBP effector structural domains that can be activated by C/EBPα [33]. For example, C/EBPα can induce PPARγ expression by recognizing the C/EBP effector structural domain of the *PPARγ* promoter. PPARγ and C/EBPα cross-regulate each other’s expression as well as governing expression of the entire adipogenicity program, which includes activation of additional transcription factors [34]. Previous studies have shown that *C/EBPα* as a transcription factor can bind to the *ACOX1* promoter region and repress its transcriptional activity [35]. This conclusion was verified in this experiment. Of these, interference with *CIDEB* significantly repressed *C/EBPα* expression. Loss of CIDEB also affected accumulation of PPARγ but to a much lesser extent, presumably reflecting its reduced positive feedback regulation by C/EBPα. It has been found that the CIDE family protein Fsp27 interacts with C/EBPβ to regulate the expression of a subset of genes downstream of *C/EBPβ* in adipocytes, and that CIDEA acts as a previously unknown transcriptional co-activator of C/EBPβ in the mammary gland to control lipid secretion and pup survival [36]. It is likely then that CIDEA causes changes in CEBPβ by regulating PPARγ and thus CIDEA. So, does CIDEB have a similar role? It has been reported in the literature that *C/EBPβ* inhibits *CIDEB* expression only under ER stress, which inhibits lipoprotein transport in the liver [37]. However, whether *CIDEB* interacts with *CEBPα* is still unclear to us.

In addition, we also found significant changes in the pyruvate metabolism after overexpression of *CIDEB* by enrichment analysis of the differential gene KEGG pathway. Here, we found that *ACOT12* was upregulated in the pyruvate metabolism pathway after overexpression of *CIDEB*. Among them, Acyl-CoA Thioesterase 12 (ACOT12), is the major cytoplasmic enzyme that preferentially hydrolyzes acetyl-CoA which hydrolyze fatty acyl-CoA to free FA and CoA and promote pyruvate metabolism [38–40]. However, acetyl-CoA is a key indicator of the regulation of metabolic status by ACOT12. Acetyl-CoA a is involved in *De novo* lipogenesis (DNL) metabolism and is used by ACC to catalyze malonyl-CoA synthesis [41]. With this change, we hypothesize that CIDEB may regulate DNL by upregulating the ACOT12 and thus changes in these pathways. Its exact mechanism needs to be verified by subsequent experiments. We also found significant changes in the malaria, taurine and hypotaurine metabolism, amphetamine addiction and cocaine addiction after overexpression of *CIDEB* by enrichment analysis of the differential gene KEGG pathway. It has been reported that lipid metabolism is significantly elevated during intraerythrocytic development of malaria parasites infected [42], which mainly through the TLR/MyD88 signaling pathway. TLR/MyD88 is a classic signaling inflammatory response that also involves in obesity development and is inhibited by the overexpression of PPARγ via the negative control of the expression of TLR4, and then activates the expression of CD36 for promoting fatty acid uptake and fat accumulation. This may highlight the important role of PPARγ in regulating.

Furthermore, we also found significant changes in the insulin signaling pathway, glycolysis/gluconeogenesis, focal adhesion and apelin signaling pathway after interference of *CIDEB* by KEGG pathway analysis. Apelin was reported to stimulate glucose uptake, increase insulin sensitivity, and regulate lipolysis and fatty acid oxidation. Apelin-13, possibly by activating the PI3K/AKT pathway, could improve the lipid metabolism [43]. PI3K/AKT is the downstream pathway of focal adhesion pathway [41], and the PI3K-AKT-mTOR signaling pathway can regulate downstream gene *SREBP1.* We found that the apelin signaling pathway also contain the cAMP-PKA pathway to regulating the expression levels of PPARγ and SREBP1. Moreover, the insulin signaling pathway controls the Glycolysis/glycogenesis pathway and mediates the interaction between proteins involved in lipid metabolism [44,45]. Therefore, we further speculated that CIDEB may regulate lipid deposition in goat intramuscular adipocytes through insulin signaling pathway, glycolysis/gluconeogenesis, focal adhesion and apelin signaling pathway by regulating the expression levels of PPARγ and SREBP1. In addition, after interfering with *CIDEB*, we also found enhanced function of the thyroid hormone signaling pathway. Studies have shown that the extract of Sanghuangporus vaninii extract can reduce blood lipids in mice, which may promote lipid metabolism and cholesterol excretion through thyroid hormone signaling pathway, and inhibit cholesterol biosynthesis [46]. We speculate that interference with *CIDEB* promotes the enhancement of thyroid hormone signaling function, promotes lipid metabolism and inhibits lipid deposition.

Moreover, two pathways are known for triglyceride metabolism within lipid droplets, cytoplasmic lipolysis (lipolysis) and lysosome-mediated autophagy (lipophagy), the latter of which involves the hydrolysis of lipid droplets by lysosomal acid lipase after engulfment by autophagic vesicles [47]. In this study we found that the lysosome pathway was altered by KEGG analysis during both overexpression and interference with *CIDEB*. Then whether CIDEB is involved in lipophagy is not clear to us, but it also gives us referable data for our next study.

In summary, the present study found that CIDEB plays an important role in intramuscular fat deposition in goats. *CIDEB* was predicted to regulate cellular lipid deposition by regulating the expression levels of *SREBP1 and PPARγ* as well as the fat synthesis-related genes *ACC* and *FASN*. Understanding the regulatory role of CIDEB in intramuscular fat deposition in goats may help improve goat meat quality.

## Figures and Tables

**Table 1:** Primers information for RT-qPCR

Gene	Full name	Sequence (5′-3′)	Tm/°C	Length/bp	Genbank ID
*PPARγ*	Peroxisome proliferator activated receptor gamma	F: AAGCGTCAGGGTTCCACTATG	60	197	NM_001285658.1
		R: GAACCTGATGGCGTTATGAGAC			
*C/EBPα*	Enhancer binding protein alpha	F: CCGTGGACAAGAACAGCAAC	60	142	XM_018062278.1
		R: AGGCGGTCATTGTCACTGGT			
*SREBP1*	Sterol regulatory element binding transcription factor 1	F: *AAGTGGTGGGCCTCTCTGA*	60	127	NM_001285755.1
		R: *GCAGGGGTTTCTCGGACT*			
*ACSL1*	Acyl-CoA synthetase long-chain family member 1	F: *TGACTGTTGCTGGAGACTGG*	60	199	XM_005698718
		R: *CAGCCGTCTTTATCCAGAGC*			
*DGAT1*	Diacylglycerol O-acyltransferase 1	F: *CCACTGGGACCTGAGGTGTC*	60	101	XM_018058728
		R: *GCATCACCACACACCAATTCA*			
*DGAT2*	Diacylglycerol O acyltransferase 2	F: CATGTACACATTCTGCACCGATT	60	100	XM_018058853.1
		R: TGACCTCCTGCCACCTTTCT			
*AGPAT6*	1-acylglycerol-3-phosphate O-acyltransferase 6	F: AAGCAAGTTGCCCATCCTCA	60	101	JI861797.1
		R: AAACTGTGGCTCCAATTTCGA			
*ATGL*	Adipose triglyceride lipase	F: *GGAGCTTATCCAGGCCAATG*	60	180	NM_001285739
		R: *TGCGGGCAGATGTCACTCT*			
*HSL*	Hormone-sensitive lipase	F: GGGAGCACTACAAACGCAACG	60	118	EU273879
		R: *TGAATGATCCGCTCAAACTCG*			
*LPL*	Lipoprotein lipase	F: *AGGACACTTGCCACCTCATTC*	60	169	XM_013966067.2
		R: *TTGGAGTCTGGTTCCCTCTTGT*			
*ACOX1*	Acyl-CoA oxidase 1	F: CGAGTTCATTCTCAACAGTCCT	60	211	NM_00103528
		R: GCATCTTCAAGTAGCCATTATCC			
*CPT1A*	Carnitine palmitoyltransferase 1A	F: TGACGGCTCTGGCACAAGAT	60	164	XM_018043311.1
		R: CGCGAAGTAGTTGCTATTCAC			
*CPTIB*	Carnitine Palmitoyltransferase 1B	F: ACGAGGAGTCTCACCACTACG	60	111	NM_001009259
		R: GTGTGAAGGACTTGTCGAACCA			
*PLIN1*	Perilipin 1	F: CCCATTGCCAGCACTTCAGA	60	95	XM_018066567.1
		R: *GCAGCGTACTCGGCAGTATCTC*			
*TIP47*	Tail-interacting protein, 47	F: GTCCGCTGACGAGACCGAA	60	319	NM_001285595.1
		R: CAGATTCTCCTCCAGTTTGTCC			
*ADFP*	Adipose differentiation-related protein/Perilipin 2	F: TTGCTGTTGCCAATACCT	60	284	NM_001285596.1
		R: CTGCATCATCCGACTTCC			
*ACSS2*	Acyl-CoA synthetase short chain family member 2	F: GGCGAATGCCTCTACTGCTT	60	100	XM_018057751
		R: GGCCAATCTTTTCTCTAATCTGCTT			
*FABP3*	Fatty acid binding protein 3	F: GATGAGACCACGGCAGATG	60	120	NM_00128570
		R: GTCAACTATTTCCCGCACAAG			
*UXT*	Ubiquitously expressed transcript	S: *GCAAGTGGATTTGGGCTGTAAC*	60	180	XM_005700842
		R: *ATGGAGTCCTTGGTGAGGTTGT*			
*CIDEB*	Cell death-inducing DFF45- like effector B	F: CAGCCGCTACCCGTCAGGAACT	60	233	Sequence obtained from the experiment
		R: CTTGCTGTGCTTGGGCTTCTCC			
*FASN*	Fatty acid synthase	F: *GGGCTCCACCACCGTGTTCCA*	60	226	NM_001285629.1
		R: *GCTCTGCTGGGCCTGCAGCTG*			
*ACC*	Acetyl-CoA carboxylase alpha	F: *CTCCAACCTCAACCACTACGG*	60	171	NM_174224.2
		R: *GGGGAATCACAGAAGCAGCC*			

RT-qPCR, quantitative real-time polymerase chain reaction; Tm, melting temperature.

**Table 2:** Screening results of *CIDEB* overexpression GO enrichment

Function classification	Pathway	Function	p-value
Biological process	GO:0015917	Aminophospholipid transport	0.015374882
Biological process	GO:0006629	Lipid metabolic process	0.040561346
Biological process	GO:0046486	Glycerolipid metabolic process	0.042506493

GO, Gene Ontology.

**Table 3:** Screening results of *CIDEB* interference GO enrichment

Function classification	Pathway	Function	p-value
Biological process	GO:0010888	Lipid transport involved in lipid storage	0.010139619
Biological process	GO:0019915	Lipid storage	0.010343653
Biological process	GO:0015917	Aminophospholipid transport	0.019999842
Biological process	GO:0071396	Cellular response to lipid	0.031200491

GO, Gene Ontology.
